# Numerical Study of Flow Boiling of ADN-Based Liquid Propellant in a Capillary

**DOI:** 10.3390/ma16051858

**Published:** 2023-02-24

**Authors:** Xuhui Liu, Gaoshi Su, Zhaopu Yao, Zhuan Yan, Yusong Yu

**Affiliations:** 1 Beijing Institute of Control Engineering, Beijing 100190, China; 2 Hydrogen Energy and Space Propulsion Laboratory, School of Mechanical, Electronic and Control Engineering, Beijing Jiaotong University, Beijing 100044, China

**Keywords:** ADN-based liquid propellant, flow boiling, microscale flow, heat reflux

## Abstract

During the operation of ADN (ammonium dinitramide, (NH^4+^N(NO_2_)^2−^))-based thrusters, the ADN-based liquid propellant, a non-toxic green energetic material, tends to flow boil in the capillary tube due to heat transfer from the wall. A three-dimensional transient numerical simulation of the flow boiling of ADN-based liquid propellant in the capillary tube was carried out using the VOF (Volume of Fluid) coupled Lee model. The flow-solid temperature and the gas–liquid two-phase distribution and the wall heat flux at different heat reflux temperatures were analyzed. The results show that the magnitude of the mass transfer coefficient of the Lee model significantly influences the gas–liquid distribution in the capillary tube. The total bubble volume increased from 0 mm^3^ to 957.4 mm^3^ when the heat reflux temperature was increased from 400 K to 800 K. The bubble formation position moves upwards along the inner wall surface of the capillary tube. Increasing the heat reflux temperature intensifies the boiling phenomenon. When the outlet temperature exceeded 700 K, the transient liquid mass flow rate in the capillary tube was already reduced by more than 50%. The results of the study can be used as a reference for the design of ADN-based thruster.

## 1. Introduction

In recent years, with the development of the economy and the increasing demand for low carbon and environmental protection, the production of high energy and green energetic materials has become a hot research issue [1]. Scientists have researched and developed new propellants and methods, promoting the development of ADN-based liquid energy materials.

ADN is a solid white salt of the ammonia cation (NH_4_^+^) and the dinitramide anion (N(NO_2_)^2−^) [2]. It melts at 91.5 °C and starts to decompose at approximately 200 °C [3]. The decomposition products and processes of ADN are quite complex, including combinations of HNO_3_, HNO_2_, N_2_O, NO_2_, NO, NH_3_, H_2_O, O, etc. A typical ADN-based liquid monopropellant is a mixture of ADN, water, and fuel (e.g., acetone, ethanol and methanol). Currently, ADN-based propellants are mainly used in the propulsion systems of microsatellites. Currently, in orbital space applications, it has been found that the propellant supply system has some problems related to capillary flow that can cause the satellite to fail. Recent experimental tests have shown that the ADN-based liquid propellant in the capillary tube would boil due to the heat reflux. This can lead to a reduction in mass flow rate and even blockage of liquid flow [4,5].

Previous studies have shown that the effect of surface tension gradually increases with decreasing channel size, and that micro-scale flow boiling can exhibit a completely different pattern to that of conventional channels. Within the micro-scale channel, the effect of local breakage and coalescence of the gas–liquid interfaces on mass and heat transfer can become significant [6]. In general, flows with tube diameters of the order of 0.1 mm are in the realm of micro-scale flows [7,8]. Flow boiling in microchannels is an extremely complex process, involving various physical phenomena such as bubble dynamics, instability of the gas–liquid two-phase flow, etc. [9]. In addition, flow boiling is also influenced by many factors such as the geometrical parameters of the channels, the surface properties of the heated wall surfaces, the physical properties of the working fluids and so on [10,11].

In the study of flow boiling in microchannels, A. Mukherjee [12] analyzed the wall heat transfer mechanism during bubble growth in microchannels under flow boiling conditions. Zhuan and Wang et al. [13] modelled the phenomenon of flow boiling in microchannels and concluded that bubble growth and coalescence were important factors in the flow pattern shift. Arvind Pattamatta et al. [14] numerically studied the conditions that promote the coalescence of Taylor bubbles within square microchannels. M. Magnini et al. [15,16] carried out numerical simulations of single elongated bubbles under flow boiling conditions in circular microchannels and calculated the local rates of mass and energy exchange at the gas–liquid phase interface. Pan et al. [17] studied the growth of vapor bubbles flowing in 2D axisymmetric microchannels under heating conditions. Matthew D. Clark et al. [18] studied the dynamic instabilities of two-phase boiling flow and discussed the critical heat flux in a microchannel by experimental method. Based on the above findings, most of the research on flow boiling in microchannels has focused on the bubble growth process of bubbles and the evolution of the gas–liquid two-phase flow pattern for regular liquids such as water, alcohol, cryogen, etc. Few simulation studies have been conducted for the boiling flow of ADN based liquid propellant in capillary tubes. Little is known about the heat transfer characteristics of this type of propellant in capillaries under the heat reflux conditions.

The objective of this paper is to investigate and elucidate the effect of heat reflux temperature (400 K~800 K) on the boiling heat transfer of ADN-based liquid propellant flow in the capillary tube through computational fluid dynamics (CFD) simulations. The ANSYS Fluent 19.2 CFD software was used for the numerical simulation to calculate the flow boiling process of ADN-based liquid propellants using the VOF coupled Lee model. In Section 2.1, the structure of the thruster and the capillary tube was presented. The second section introduces the initial and boundary conditions, and the numerical methods. In Section 2.3, the mathematical models describing the flow, heat transfer, and phase change were described. In Section 3, the effect of the mass transfer coefficient, the constant coefficient of the Lee model, on the flow boiling process was discussed. Then the effects of the heat reflux temperature on the heat transfer and two-phase flow were analyzed in Section 3.2 and Section 3.3, respectively.

## 2. Mathematical Models and Validation

### 2.1. Physical Model

**Figure 1:**
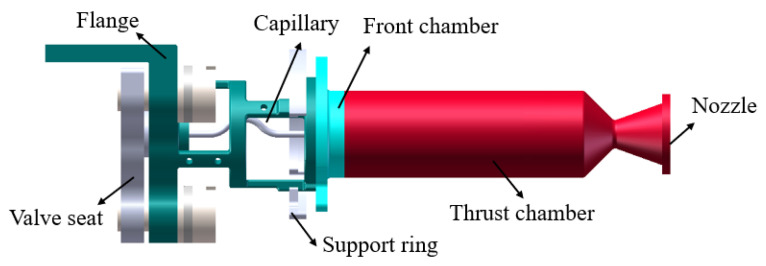
Geometric model of the ADN based thruster.

The ADN-based thruster consists mainly of a valve seat, flange, support ring, capillary tube, front chamber, thrust chamber, and nozzle, the geometric model of which is shown schematically in Figure 1. During operation of the thruster, the liquid propellant in the upstream capillary tube is affected by the exothermic catalytic combustion in the downstream thrust chamber, resulting in a gas–liquid two-phase flow phenomenon with local boiling.

**Figure 2:**
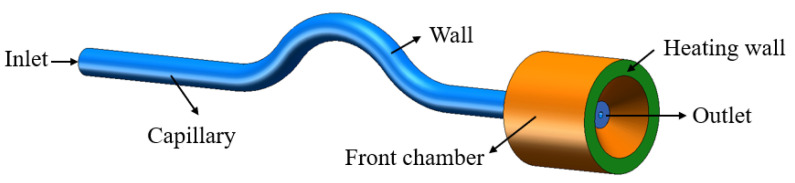
Geometric schematic diagram with boundary conditions of the capillary tube.

Figure 2 shows a schematic diagram of the geometric model with boundary conditions. To reduce the number of computations, in this study, the capillary tube and part of the front chamber structure are used as the computational domain, with the pressure inlet on the left side of the capillary tube and the pressure outlet on the right side, and the heating wall is set as a constant wall temperature boundary.

**Figure 3:**
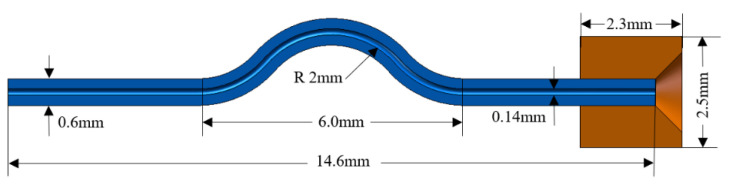
Schematic representation of the structural geometry of the capillary tube and part of the front chamber.

The dimensional parameters of the geometric model of the capillary tube are given in Table 1, with an inner diameter of 0.14 mm and an outer diameter of 0.6 mm. Figure 3 shows a schematic diagram of the geometry of the capillary tube and part of the front chamber of the structure.

**Figure 4:**
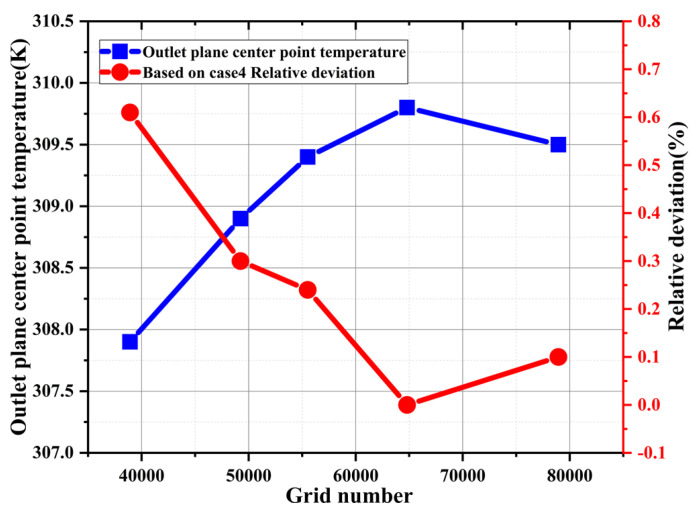
Comparison of center point temperature and relative deviations of the capillary tube exit plane with different grid cell numbers.

Before running the simulation, the grid independence is first verified, and the effect of the grid number on the simulation accuracy is shown in Figure 4; the temperature at the center point of the capillary tube outlet plane at different grid numbers is used as the calculation criterion. The results show that the temperature at the center of the capillary tube outlet plane is gradually stabilized with the gradual increase in the grid number. Among them, the calculation results under the grid number of 64,801 are used as the benchmark, and the relative deviations for the grid number of 55,512 and 78,939 are 0.24% and 0.10% respectively, which are both within the relative deviation of 0.30%. This shows that the case with the grid number of 55,512 could meet the calculation requirement.

### 2.2. Simulational Conditions and Numerical Methods

In this study, the following assumptions were made on the premise that the boiling process of the ADN-based liquid propellant flow in the capillary tube can be accurately described: (1) the flow state of the ADN-based liquid propellant in the capillary tube was determined to be turbulent flow; (2) the ADN-based liquid propellant gas–liquid two-phase flow was determined to be an incompressible flow; (3) the surface tension between the gas and liquid phases was considered.

This calculation assumes that the initial temperature of the computational domain is 300 K. The capillary tube is filled with ADN-based liquid propellant and the fluid in the fluid domain is still. Assuming that the properties of the ADN-based liquid propellant are constant. The properties of the ADN-based liquid propellant are given in Table 2. The turbulence model is selected from the standard k−ε model. A variable time-step of 10^−5^~10^−6^ s and a global Courant number (uΔt/Δc) of unity are used to ensure numerical stability. The numerical details and discretization methods are given in Table 3.

### 2.3. Mathematical Models

In order to better simulate the variation of the flow pattern of ADN-based liquid propellant in the capillary tube, the VOF model [20] was used in this study to simulate the gas–liquid two-phase flow of ADN-based liquid propellant in the capillary tube. The Continuity equations for the gas and liquid phases are

(1) $$\frac{\partial }{\partial t}\left(\alpha_{l}\rho_{l}\right)+\nabla \cdot \left(\alpha_{l}\rho_{l}V\right)=-S_{M}$$

(2) $$\frac{\partial }{\partial t}\left(\alpha_{v}\rho_{v}\right)+\nabla \cdot \left(\alpha_{v}\rho_{v}V\right)=S_{M}$$

where $\alpha_{l}$ and $\alpha_{v}$ are the volume fractions of the liquid and gas phases, respectively. $\rho_{l}$ and $\rho_{v}$ are the densities of the liquid and gas phases, respectively, in $\mathrm{kg}\cdot m^{-3}$; V is the fluid velocity in $m\cdot s^{-1}$; and $S_{M}$ is the boiling phase change mass of the liquid subjected to wall heating in $\mathrm{kg}\cdot m^{-3}\cdot s^{-1}$.

The momentum and energy conservation equations for gas–liquid two-phase flow under the VOF model are similar to those for single-phase flow, and the expressions for the momentum equation are shown below:

(3) $$\frac{\partial }{\partial t}\left(\rho V\right)+\nabla \left(\rho VV\right)=-\nabla P+\nabla \cdot \left[\mu \left(\nabla V+\nabla V^{T}\right)\right]+\rho g+F$$

(4) $$\rho =\alpha_{l}\rho_{l}+\alpha_{v}\rho_{v}$$

where V is the fluid velocity in $m\cdot s^{-1}$; P is the fluid pressure in Pa; μ is the fluid dynamic viscosity in Pa⋅s; T is the temperature in K; g is the acceleration of gravity in $m\cdot s^{-2}$; and F is the surface tension per unit volume of fluid in $N\cdot m^{-3}$.

The expression of the energy equation is shown below:

(5) $$\frac{\partial }{\partial t}\left(\rho E\right)+\nabla \left[V\left(\rho E+\rho \right)\right]=\nabla \left(\lambda \nabla T\right)+L_{h}$$

(6) $$\lambda =\alpha_{l}\lambda_{l}+\alpha_{v}\lambda_{v}$$

(7) $$E=\frac{\alpha_{l}\rho_{l}E_{l}+\alpha_{v}\rho_{v}E_{v}}{\alpha_{l}\rho_{l}+\alpha_{v}\rho_{v}}$$

where $L_{h}$ is the energy source term, which means the latent heat of evaporation generated by the thermal phase change in ADN-based liquid propellant in $W\cdot m^{-3}$; λ is the effective thermal conductivity, $\lambda_{l}$ and $\lambda_{v}$ are the thermal conductivity of liquid and gas phases, respectively, in W/(m⋅K); $E_{l}$ and $E_{v}$ are the energy of liquid and gas phases, respectively, in J/kg; *T* is the temperature in K.

The phase change evaporation of the ADN-based liquid propellant in the capillary tube is simulated using the Lee model [21]. In the Lee model, the liquid–gas mass transfer (evaporation and condensation) is controlled by the vapor transport equation.

(8) $$\frac{\partial }{\partial t}\left(\alpha_{v}\rho_{v}\right)+\nabla \cdot \left(\alpha_{v}\rho_{v}{\vec{V}}_{v}\right)={\dot{m}}_{lv}-{\dot{m}}_{vl}$$

The Lee model defines positive mass transfer as the mass transfer from liquid to vapor. The specific expression is shown below.

If $T_{l}>T_{sat}$ (evaporation):

(9) $${\dot{m}}_{lv}=\mathrm{co}eff*\alpha_{l}\rho_{l}\frac{\left(T_{l}-T_{sat}\right)}{T_{sat}}$$

If $T_{v}<T_{sat}$ (condensation):

(10) $${\dot{m}}_{vl}=\mathrm{co}eff*\alpha_{v}\rho_{v}\frac{\left(T_{sat}-T_{v}\right)}{T_{sat}}$$

where $T_{sat}$ is the saturation temperature of the ADN-based liquid propellant in K, and coeff is the time factor characterizing the phase change hysteresis, which can be interpreted as the relaxation time in 1/s.

In this study, the surface tension between the gas–liquid interface of ADN-based liquid propellants is considered using the CSF (continuum surface force) model proposed by Brackbill et al. [22]. Its expression is shown as follows:

(11) F≈σκ∇F

(12) n=−∇F

(13) $$\kappa =\nabla \cdot \left(\frac{n}{\left|n\right|}\right)$$

where F is the surface tension at the gas–liquid interface in N; σ is the surface tension coefficient in N/m, which is assumed to be constant and does not vary with temperature; κ is the radius of curvature at the gas–liquid interface; and n is the normal unit vector at the gas–liquid interface.

When heat from the combustion chamber is transferred through the wall to the upstream capillary tube region, there are physical phenomena such as heat conduction within the solid part of the capillary tube; fluid–solid coupling heat transfer and heat radiation from the solid to the environment occur. This heat transfer process is the basis for numerical simulations to obtain accurate boiling results for the flow in the capillary tube.

The energy equation for heat transfer within the solid is shown as follows [23]:

(14) $$\rho_{s}c_{s}\frac{\partial T}{\partial t}=k_{s}\nabla^{2}T+{\dot{S}}_{r}$$

where $\rho_{s}$ is the density of the solid (capillary tube, front chamber) in $\mathrm{kg}\cdot m^{-3}$; $c_{s}$ is the specific heat capacity of the solid in J/(kg⋅K); *T* is the temperature of the solid in K; $k_{s}$ is the thermal conductivity of the solid in W/(m⋅K); ${\dot{S}}_{r}$ is the radiation heat source.

At the fluid–solid coupling interface, the fluid–solid heat flow conservation should be satisfied [24]:

(15) $${K_{cond}\frac{\partial T}{\partial n}|}_{wf}=q^{conv}=h_{conv}\left(T_{f}-T_{w}\right)$$

where $K_{cond}$ is the thermal conductivity of the solid in W/(m⋅K); $q^{conv}$ is the heat exchange volume; $h_{conv}$ is the local convective heat transfer coefficient in $W/\left(m^{2}\cdot K\right)$; $T_{f}$ is the fluid temperature in K; $T_{w}$ is the wall temperature, in K.

The P1 radiation model [25] is used in this study to account for the radiative heat exchange between the solid and the external space:

(16) $$q_{r}=-\Gamma \nabla G$$

(17) $$\Gamma =\frac{1}{3\left(a+\sigma_{s}\right)-C\sigma_{s}}$$

where $q_{r}$ is the radiation flux in W; a is the absorption coefficient; $\sigma_{s}$ is the scattering coefficient; G is the incident radiation; and C is the linear anisotropic phase function coefficient.

The transmission equation is shown below:

(18) $$\nabla \cdot \left(\Gamma \nabla G\right)-aG+4an^{2}\sigma T^{4}=S_{G}$$

where *n* is the refractive index of the medium; σ is the Stefan–Boltzmann constant; and $S_{G}$ is the radiation source term.

The final expression of the P1 radiation model is obtained by combining the above equations.

(19) $$-\nabla \cdot q_{r}=aG-4an^{2}\sigma T^{4}$$

**Figure 5:**
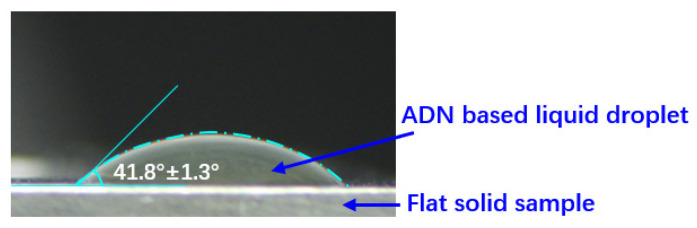
The image of the drop was captured by a high-resolution camera.

The static contact angle for wall adhesion calculation in ANSYS Fluent was determined using the sessile drop, the most commonly used contact angle measurement method [26]. The ADN-based liquid drop was placed on the flat sample that is made of the same material as the capillary tube. The image of the drop was captured by a high-resolution camera (see Figure 5). After six repetitive shots and tests, the contact angle was determined to be 41.8° ± 1.3° under current conditions.

### 2.4. Model Validation

**Figure 6:**
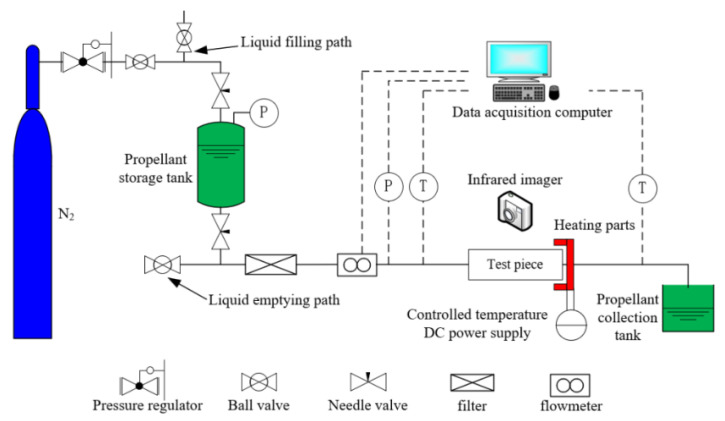
Schematic diagram of the experimental system.

In order to verify the accuracy of the established numerical model, numerical simulations of the flow boiling of ADN-based liquid propellant in the capillary tube were performed at a heat reflux temperature of 800 K, and the capillary inlet and outlet pressure drop of 0.5 MPa. The simulated inlet mass flow rate and the temperature at the outer wall surface of the capillary tube (at about 4.3 mm from the capillary tube outlet) were compared with the experimental results. The same capillary structure and boundary conditions were used for the simulation and the experiment. The experimental system (Figure 6) consists of three parts: a storage tank, test section bench, and data acquisition system. The storage tank contains ADN-based liquid propellant, and a constant pressure nitrogen cylinder is used to control the inlet pressure value. The detailed experimental system and descriptions are given in [4].

Table 4 shows the results of the comparison between the numerical simulation and the experimental results. The results showed that the experimental and simulated capillary tube inlet mass flow rates were 0.0308 g/s and 0.0300 g/s, respectively (with an error of about 5.3%). The experimental and simulated temperatures of the outer capillary tube wall surface (at approximately 4.3 mm from the capillary tube outlet) were 398.7 K and 420.1 K, respectively (with an error of approximately 2.6%). The total mass flow rate of ADN-based propellant at the inlet and outlet of the capillary tube was calculated to be 0.0300 g/s and 0.0298 g/s, respectively, with an inlet and outlet flow rate deviation of 6.7‰, indicating that the two-phase calculation of the current simulation is conserved.

The relative error of the temperature of the outer wall surface at a distance of approximately 4.3 mm from the capillary tube outlet is:

(20) $$\frac{\left|420.1-398.7\right|}{398.7}\times 100\%\approx 5.3\%$$

The relative error of the capillary tube inlet mass flow rate is:

(21) $$\frac{\left|0.030-0.038\right|}{0.038}\times 100\%\approx 2.1\%$$

## 3. Results and Discussion

### 3.1. Analysis of the Effect of Mass Transfer Coefficient on Flow Boiling

In this study, the Lee model is used to simulate the flow boiling process of ADN-based liquid propellant in the capillary tube, and the specific expressions of the Lee model can be seen in Equations (2)–(10). The Lee model involves the problem of taking the value of the mass transfer coefficient. In general, the value of the mass transfer coefficient ranges from 0.1 to 10^7^ s^−1^, and the value of the mass transfer coefficient is related to the grid size, time step, specific phase transition phenomena, and other factors [27]. The mass transfer coefficient has been taken as 100 in some studies [28,29,30], while in others it was taken as 0.1 [31,32,33]. Therefore, in this study, in order to determine the appropriate mass transfer coefficient, the effect of the phase transition mass transfer coefficient on the boiling of the flow in the capillary tube was first numerically simulated.

**Figure 7:**
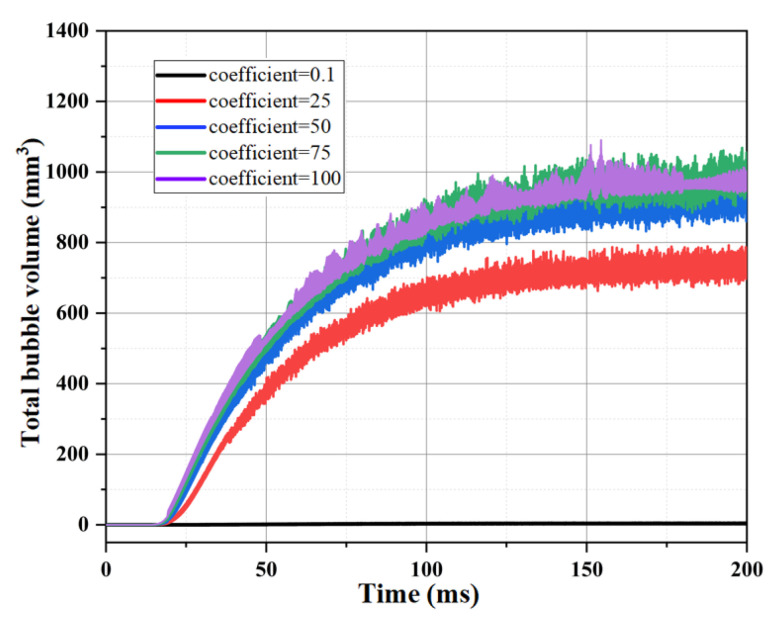
Comparison of bubble volumes in capillary tube at different mass transfer coefficients.

Figure 7 compares and analyzes the variation of the total volume bubbles in the capillary tube at different mass transfer coefficients (0.1, 25, 50, 75, 100). As can be seen from the figure, the value of the mass transfer coefficient affects the degree of vaporization of the ADN-based liquid propellant flow boiling in the capillary tube under the same simulated conditions, and the total volume of bubbles generated in the capillary tube increases with the increase in the mass transfer coefficient. When the mass transfer coefficient was 0.1, the total bubble volume was about 4.0 mm^3^; whereas, when the mass transfer coefficient was 100, the total bubble volume generated reached about 953 mm^3^.

**Figure 8:**
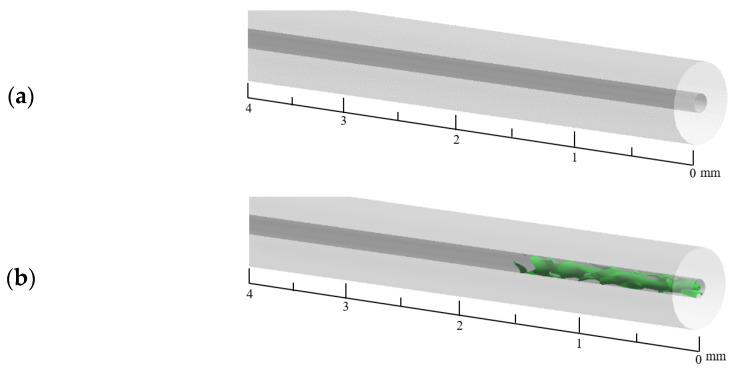

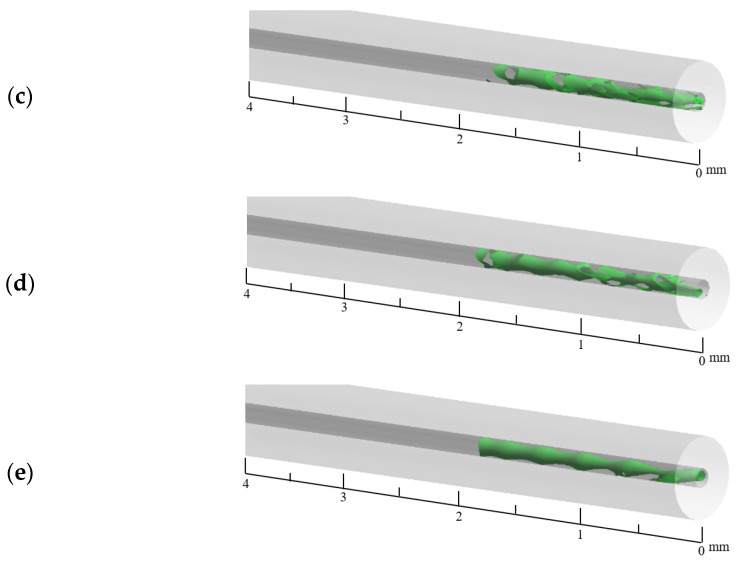
Gas–liquid iso-surface in a capillary tube at different mass transfer coefficients: (**a**) 0.1 s^−1^, (**b**) 25 s^−1^, (**c**) 50 s^−1^, (**d**) 75 s^−1^, and (**e**) 100 s^−1^.

Figure 8 shows the plot of the gas–liquid iso-surface area in the capillary tube for different mass transfer coefficients at 200 ms. It can be seen from the figure that the magnitude of the mass transfer coefficient has a certain effect on the gas–liquid iso-surface of the €-based liquid propellant in the capillary tube. The smaller the mass transfer coefficient, the more fragmented the gas–liquid iso-surface appears. The larger the mass transfer coefficient, the more complete the gas–liquid iso-surface is. The reason for this phenomenon is that the mass transfer coefficient affects the vaporization degree €ADN-based liquid propellant. When the mass transfer coefficient is small, there is less vapo€ed ADN-based liquid propellant in the capillary tube and more tiny bubbles are scattere€ on the inner wall of the capillary tube. As the mass transfer coefficient increases, the vaporization rate is accelerated, and the initially tiny bubbles gradually grow and coalesce.

Combined with the literature, it is known that for the flow boiling problem, Z. Yang et al. [34] proposed to set the mass transfer coefficient to 100 s^−1^, which means that the accuracy of the simulation can be well maintained, and a better convergence can be ensured.

### 3.2. Analysis of Influence of Heat Reflux Temperature on Temperature Distribution

In this study, the phase change heat transfer process of ADN-based liquid propellant in the capillary tube is investigated at different heat reflux temperatures, and five numerical simulation conditions are shown in Table 5.

**Figure 9:**
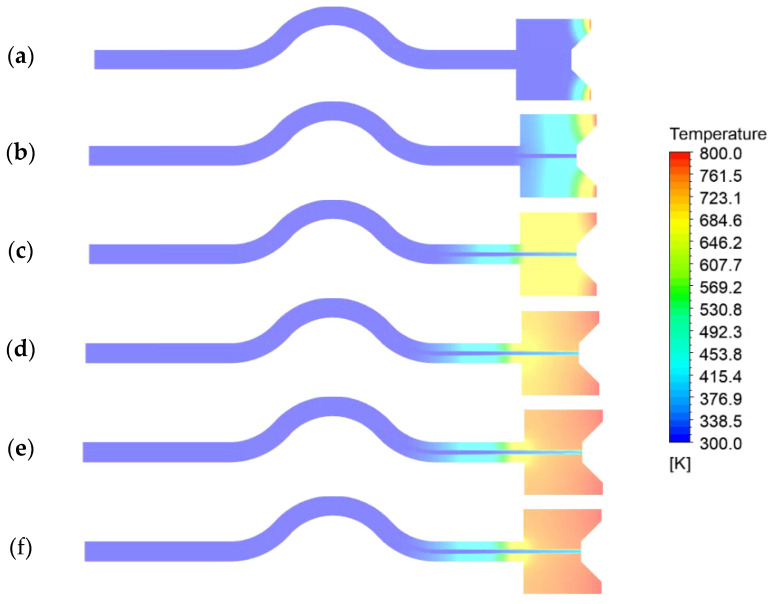
Temperature distribution in the axial section of the capillary tube at different times: (**a**) 0 ms, (**b**) 10 ms, (**c**) 50 ms, (**d**) 100 ms, (**e**) 150 ms, and (**f**) 200 ms.

Figure 9 shows the contour of the temperature distribution of the axial section of the capillary tube at different moments (0~200 ms). As can be seen from the figure, when the heat reflux temperature is 800 K, the heat is gradually transferred upstream under the effect of heat conduction in the front chamber and the capillary tube solid. During the upstream transfer, the temperature distribution of the front chamber and the outer wall surface of the capillary tube is uniform. At 100 ms, the temperature of the outer surface of the front chamber is essentially maintained at about 771 K, and the temperature of the outer surface remains essentially constant with the time delay. Measurements found 100 ms, from the capillary tube outlet 4.6mm at the outer wall temperature of 340 K, 200 ms temperature of 348 K, the temperature increases slowly. Analysis of the reason may be due to the place reached near the bend, the capillary tube ADN-based liquid propellant flows through the place, the flow rate increased, making it more difficult to further transfer the heat to the upstream, so that the temperature change in the outer surface of the capillary tube is relatively slow.

**Figure 10:**
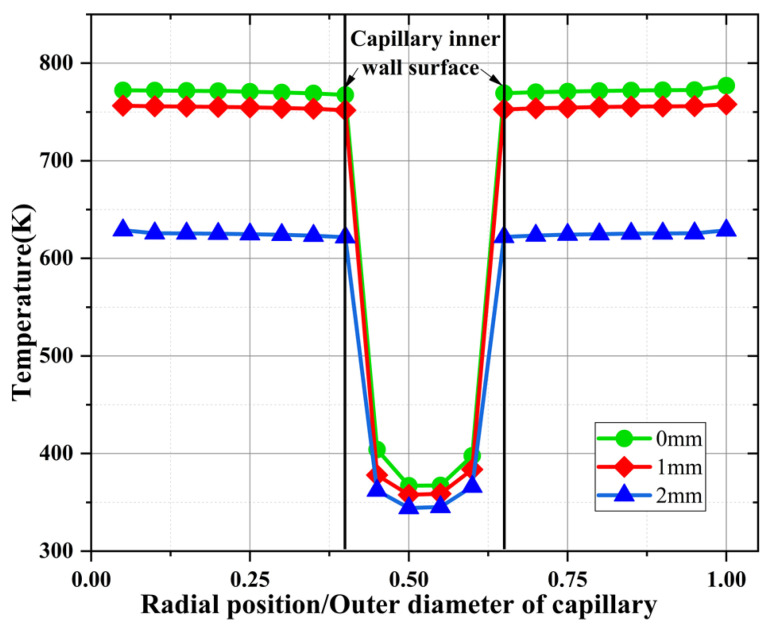
Capillary tube radial temperature distribution at 800 K.

Figure 10 shows the radial temperature distribution of the capillary tube at 0 mm, 1 mm, and 2 mm from the capillary exit at a temperature of 800 K for 200 ms. The horizontal coordinate is the ratio of the radial position of the capillary to the outer diameter of the capillary. As can be seen from the figure, the capillary tube in the same section, the temperature shows an axisymmetric distribution pattern; the temperature of the solid part of the capillary tube is basically the same at 200 ms. Under the cooling effect of the ADN-based liquid propellant, the temperature of the inner wall surface of the capillary tube was found to decrease rapidly, and the closer to the center region of the capillary tube, the lower the temperature. From this figure, it can be seen that when the heat reflux temperature is transferred to the inner wall surface of the capillary tube, the ADN-based liquid propellant first generates bubbles on the inner wall surface under the effect of fluid–solid coupling heat transfer, so that the phase change in the ADN-based liquid propellant can be suppressed by reducing the heat transfer to the inner wall surface.

**Figure 11:**
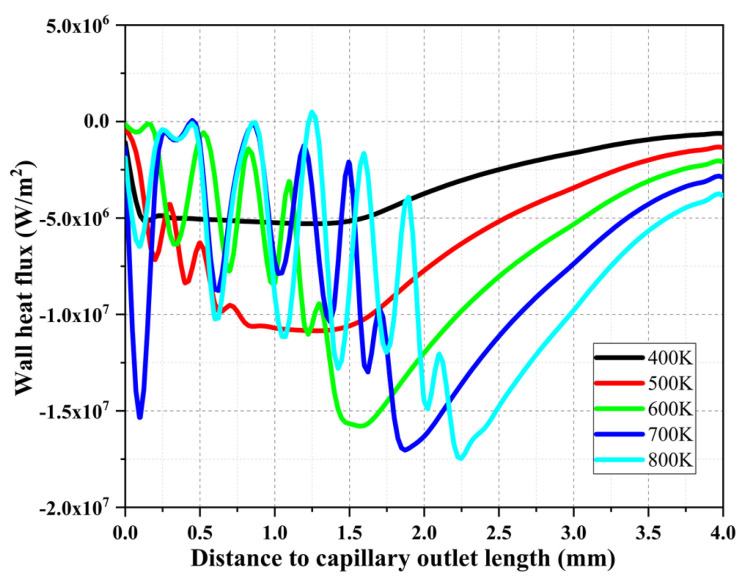
Comparison of wall heat flux in the capillary tube for different heat reflux temperatures.

Figure 11 shows the comparison of the wall heat flux of the capillary tube at different heat reflux temperatures (400 K~800 K) and the spectral analysis. As can be seen from the figure, with the gradual increase in the heat reflux temperature, the fluctuation of the wall heat flux becomes larger, indicating that the increase in the heat reflux temperature enhances the heat exchange at the wall surface. When the heat reflux temperature is above 600 K, it is found that the wall heat flux along the upstream direction of the capillary tube has an obvious inflection point, combined with the temperature change trend in Figure 8. It can be judged that the inflection point location of the wall heat flux is the starting point of flow boiling of ADN-based liquid propellant. In addition, the location of the inflection points gradually moved upstream, increasing from 1.50 mm at 600 K to 2.25 mm at 800 K.

**Figure 12:**
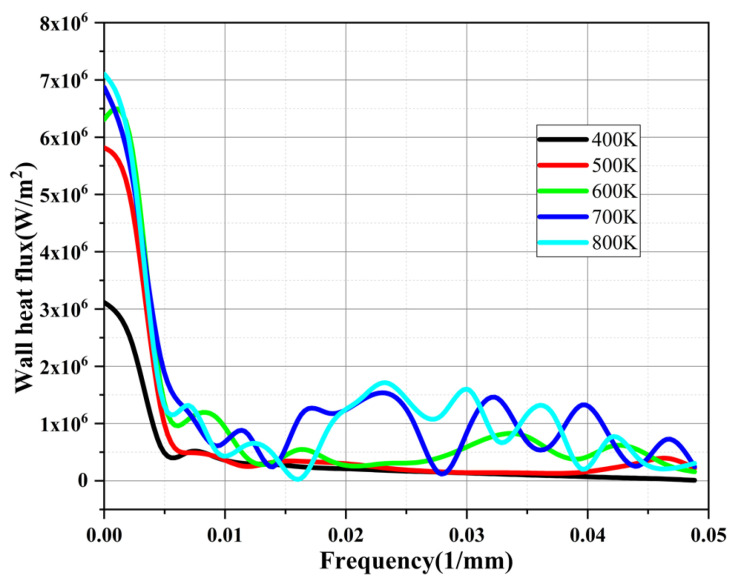
FFT analysis of wall heat flux in the capillary tube for different heat reflux temperatures.

Figure 12 shows the FFT (Fast Fourier Transform) analysis results of the wall heat flux for different heat reflux temperatures, with increase in frequency; that is, the closer to the capillary tube outlet, the more the amplitude of the wall heat flux gradually increases. When the heat reflux temperature is 800 K, the maximum amplitude is 7 × 10^6^ W/m^2^. When the heat reflux temperature is 400 K, the maximum amplitude of the wall heat flux is 3 × 10^6^ W/m^2^. The amplitude is reduced by about 57%. As the heat reflux temperature gradually increases, more bubbles are generated by the phase transition of ADN-based liquid propellants in the capillary tube. As a result, the amplitude of the wall heat flux in the capillary tube gradually increases as the bubbles form and coalesce. Additionally, with the gradual increase in frequency, the amplitude of the wall heat flux density shows a tendency to gradually decrease.

### 3.3. Analysis of Heat Reflux Temperature on Gas–Liquid Two-Phase Flow

**Figure 13:**
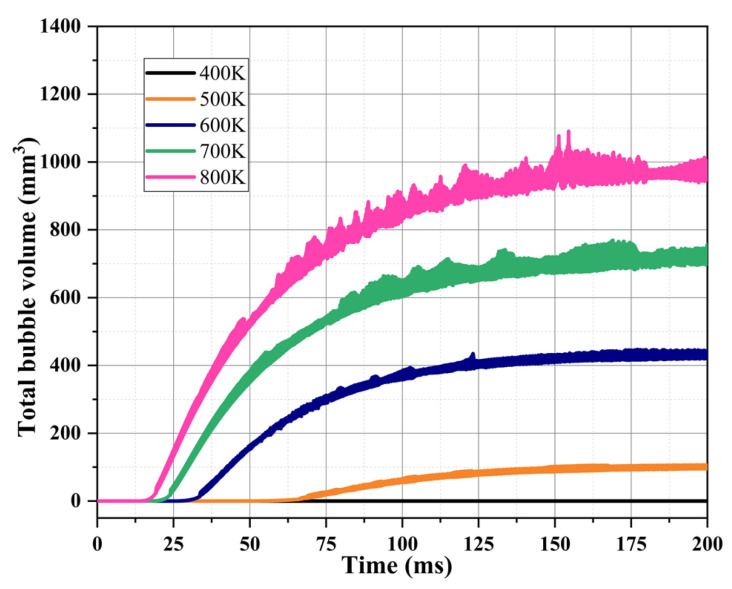
Comparison of bubble volumes in capillary tube at different heat reflux temperatures.

Figure 13 shows the variation of the total volume of bubbles generated in the capillary tube at different heat reflux temperatures. In can be seen from the figure that with the gradual increase in the heat reflux temperature (from 400 K to 800 K), the volume of the bubbles formed in the capillary tube increases significantly, indicating that the two-phase flow in the capillary tube is more intense. It is speculated that this may be due to the higher heat reflux temperature enhancing the fluid–solid heat transfer in the capillary tube section, causing the bubble formation zone to move upstream, resulting in a phase change in more ADN-based liquid propellant in the capillary tube.

**Figure 14:**
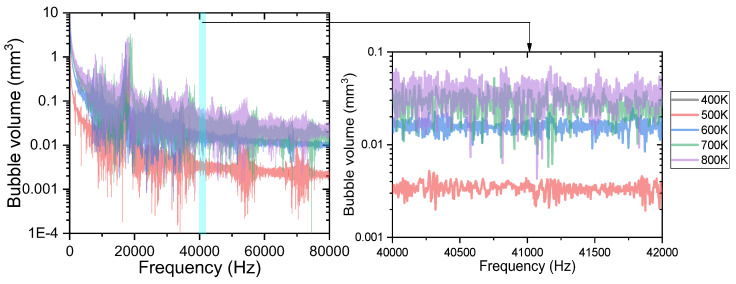
FFT analysis of bubble volumes in capillary tube at different heat reflux temperatures.

Figure 14 shows the FFT analysis of the bubble volume in the capillary tube versus time. As can be seen from the figure, the fluctuation frequencies of the bubble volume under different heat reflux temperatures are all around 17 kHz. The fluctuation range of the bubble volume in the capillary tube increased as the heat reflux temperature increased. When the heat reflux temperature is 400 K, the phase transition temperature of the ADN based liquid propellant is not reached. Therefore, at 400 K, the single-phase flow in the capillary tube is mainly liquid phase, and the bubble volume does not fluctuate significantly. As the heat reflux temperature increases, the ADN liquid propellant in the capillary tube changes phase, and the bubble volume fluctuates significantly with the formation and coalescence of bubbles. When the fluctuation frequency is 17 kHz, the fluctuation range of the bubble volume increases from 0.04 mm^3^ at 500 K to 1.83 mm^3^ at 800 K.

**Figure 15:**
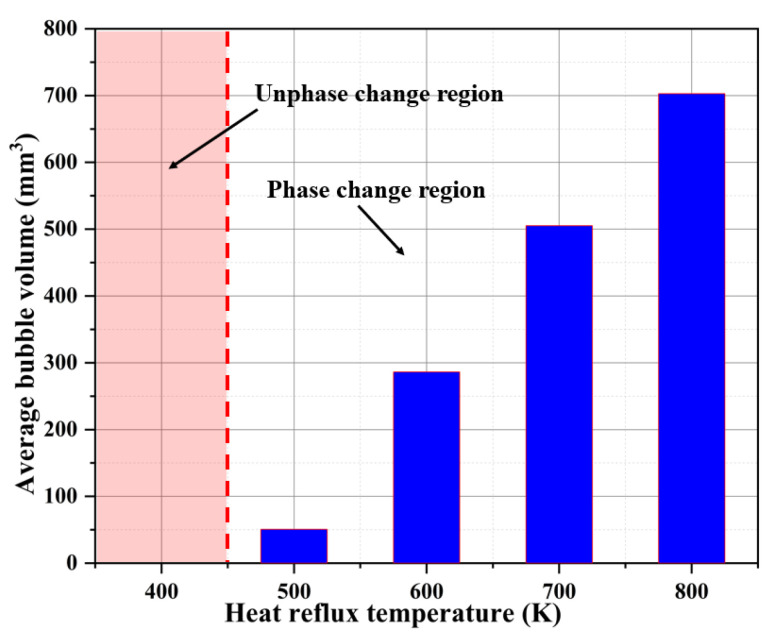
Comparison of average bubble volumes in capillary tube at different heat reflux temperatures.

Figure 15 shows the comparison of the average bubble volumes inside the capillary tube and the time of bubble generation. From the figure, it can be seen that with the gradual increase in the heat reflux temperature (400 K~800 K), the average bubble volumes generated in the capillary tube gradually increase (0 mm^3^, 50 mm^3^, 286 mm^3^, 505 mm^3^, 702 mm^3^), and the increase in the heat reflux temperature intensifies the phase change in the ADN-based liquid propellant in the capillary tube, so that more bubbles are generated in the unit time.

**Figure 16:**
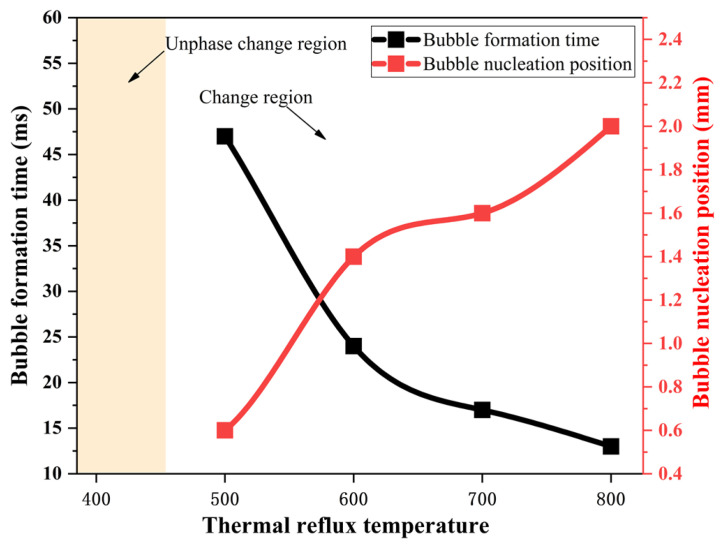
Comparison of bubble generation times and bubble formation position at different heat reflux temperatures.

Figure 16 shows the comparison of the bubble generation times and bubble formation positions at different heat reflux temperatures. From the figure, it can be seen that bubbles are generated earlier in the capillary tube at higher heat reflux temperatures, and the first bubble is generated at 47 ms for the ADN-based liquid propellant at 500 K, while the time of the first bubble generation is advanced to 13ms when the temperature reaches 800 K.

Combined with the bubble formation position curve analysis, it was found that with the gradual increase in the heat reflux temperature, the bubble formation area in the capillary tube gradually moved upstream. When the thermal immersion temperature is 400 K, the saturation temperature of ADN-based liquid propellant in the capillary tube flow state has not been reached; therefore no phase change at this temperature, the flow state for the ADN-based liquid propellant based single-phase flow; when the heat reflux temperature exceeds 500 K, there will be a phase change, bubble formation zone from the capillary tube outlet gradually moved upstream. The bubble formation zone moves from 0.6 mm at 500 K to 2.0 mm at 800 K (distance from the capillary tube outlet length).

**Figure 17:**
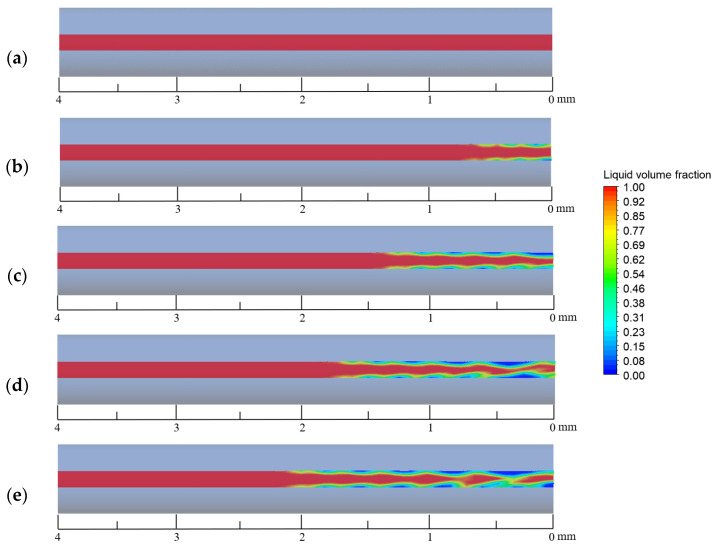
Axial gas–liquid distribution in capillary tube at different heat reflux temperatures: (**a**) 400 K, (**b**) 500 K, (**c**) 600 K, (**d**) 700 K, and (**e**) 800 K.

In order to clarify the effect of the heat reflux temperature on the gas–liquid two-phase flow distribution in the capillary tube, the characteristics of the gas–liquid phase distribution in the capillary tube at 200 ms were analyzed, as shown in Figure 17. At a temperature of 400 K, there is no phase change in the capillary tube due to the low temperature, so the flow pattern at this time is a single-phase flow of ADN-based liquid propellant. At a temperature of 500 K, the ADN-based liquid propellant generates bubble formation zones on the downstream inner wall surface of the capillary tube through fluid-solid coupling heat transfer. With further increase in temperature, the bubble formation zone moves upstream and merging between the bubbles occurs at 800 K. At 400 K, no phase change occurred and from 500 K to 800 K, the bubble formation zone was 0.6 mm, 1.4 mm, 1.6 mm, and 0.6 mm from the capillary tube outlet in the order of length.

**Figure 18:**
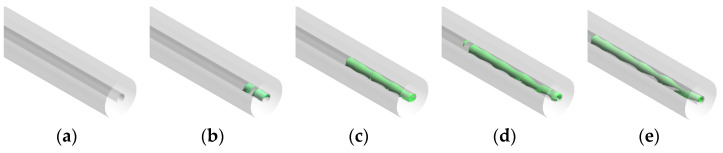
Gas–liquid iso-surface in capillary tube at 200 ms at different heat reflux temperatures: (**a**) 400 K, (**b**) 500 K, (**c**) 600 K, (**d**) 700 K, and (**e**) 800 K.

Figure 18 shows the ratio of the gas–liquid iso-surface area in the capillary tube at different heat reflux temperatures. From the figure it can be seen that the gas–liquid iso-surface in the capillary tube gradually develops as the heat reflux temperature increases. At 400 K no phase change is produced, and the flow is single-phase at 200 ms. When it is greater than 400 K, the heat transfer to the capillary tube due to the uniform temperature of the heat return dips, making the flow pattern in the capillary tube for the annular flow. With the gradual increase in the heat reflux temperature, the bubbles in the capillary tube gradually grow and merge when the flow pattern changes from the circular flow to wave flow.

**Figure 19:**
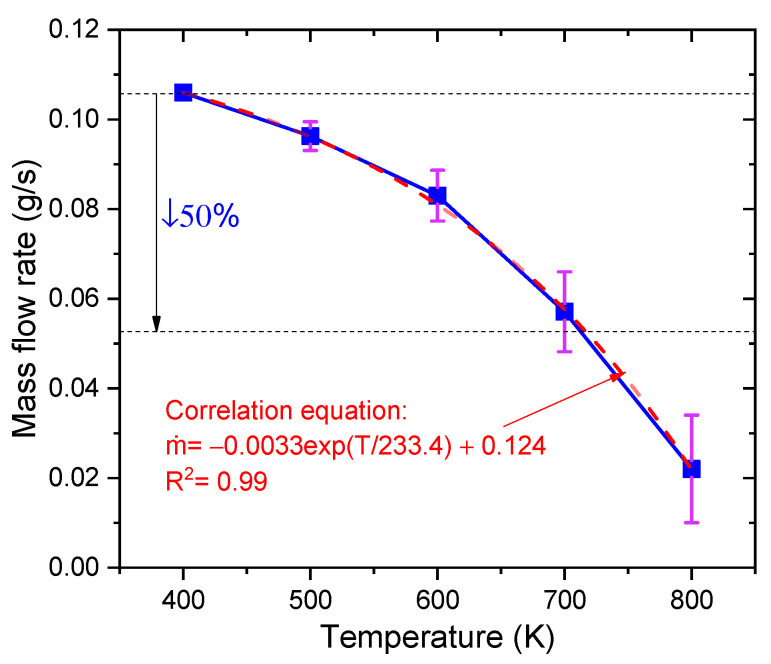
The liquid mass flow rate at five different heat reflux temperatures ranging from 400 K to 800 K. The blue solid line is the average liquid mass flow rate within 200 ms. The red dashed line is the fit result.

Figure 19 shows the liquid mass flow rate at five different heat reflux temperatures. The blue solid curve value is the average liquid mass flow rate within 200 ms. The width of error bars represents the fluctuation amplitude of the transient liquid mass flow rate. According to the results, the liquid mass flow rate decreases rapidly from 0.106 g/s to 0.022 g/s with increasing the heat reflux temperature (from 400 K to 800 K). Due to the increased instability of the gas–liquid flow at high heat reflux temperatures, the amplitude of the transient liquid mass flow increases. When the outlet temperature exceeds 700 K, the transient liquid mass flow rate in the capillary tube was already reduced by more than 50%. Such a large drop in the ADN-based propellant mass flow rate should be avoided in practical applications, so the heat reflux temperature must be controlled within 700 K. Finally, the equation for the correlation (see Figure 19) between the heat reflux temperature and the liquid mass flow rate was determined by the fitting method.

## 4. Conclusions

In this study, the effect of mass transfer coefficient in Lee model on ADN-based liquid propellant flow boiling is analyzed by numerical simulation for the first time, and the mass transfer coefficient applicable to ADN-based liquid propellant flow boiling is determined. The heat reflux immersion temperature is revealed for the gas–liquid two-phase flow and the temperature distribution law in capillary tube, which has some guidance for the design of ADN-based thrusters. The main conclusions can be drawn as follows.

(1) Simulation of the flow boiling of ADN-based liquid propellant in the capillary tube can be achieved by using the VOF-coupled Lee model. The inlet mass flow rate is about 0.0300 g/s, with an error of about 5.3% from the experimental result (0.0308 g/s), and the outer surface temperature at about 4.3 mm from the capillary tube outlet is about 420.1 K, with an error of about 2.6% from the experimental result (398.7 K), which meets the accuracy requirement;
(2) The mass transfer coefficient in the Lee model affects the frequency of bubble generation in the capillary tube under the same simulation conditions. As the mass transfer coefficient gradually increases (from 0.1 to 100), more volume bubbles are generated in the capillary tube (from 4.0 mm^3^ to 953.0 mm^3^), and the gas–liquid equivalence surface gradually becomes complete. For the flow boiling problem of ADN-based liquid propellant in the capillary tube, the mass transfer coefficient is taken as 100 s^−1^ to maintain the accuracy of the simulation well and to ensure a good convergence;
(3) During the transfer of the heat reflux temperature upstream, the temperature of the front chamber and the outer wall surface of the capillary tube are uniformly distributed, and the temperature is symmetrically distributed along the axial direction. Due to the cooling effect of the ADN-based liquid propellant, the temperature of the inner wall surface of the capillary tube is slightly lower than that of the outer surface;
(4) The increase in the heat reflux temperature (from 400 K to 800 K) intensified the flow boiling in the capillary tube, and the average bubble volumes in the capillary tube increased significantly (from 0 mm^3^ to 702 mm^3^). The time of the first bubble formation is gradually advanced, and the bubble formation position moves upstream;
(5) As the heat reflux temperature increases, the local boiling phenomenon of the ADN-based liquid propellant in the capillary gradually intensifies. When the outlet temperature exceeded 700 K, the transient liquid mass flow rate in the capillary was already reduced by more than 50%. In the design of the thruster structure, technologies such as adding insulating layers or thermal coatings to the capillary outlet are used to ensure that the temperature at the capillary outlet area does not exceed 700 K;
(6) In this study, the ADN-based liquid propellant physical parameters are assumed to remain constant. In fact, the physical parameters change with temperature. The physical parameters (density, specific heat, viscosity, surface tension, etc.) of ADN-based liquid propellants at different temperatures will be measured experimentally in later studies to verify the model in this paper in detail.

## Figures and Tables

**Table 1:** The geometry model parameters.

	Parameter	Value
Capillary tube	Inner diameter (mm)	0.14
	Out diameter (mm)	0.60
	Horizontal length (mm)	14.60
Front chamber	Inner diameter (mm)	0.60
	Out diameter (mm)	2.50
	Horizontal length (mm)	2.30

**Table 2:** Physical properties of the ADN-based liquid propellant [4,19].

Parameters	Value
Density (kg/m^3^)	1550
Cp (specific heat) (J/kg·K)	2350
Thermal conductivity (W/m·K)	0.8
Viscosity (kg/m-s)	0.0046
Molecular weight (kg/kmol)	124
Reference temperature (K)	298.15

**Table 3:** Numerical details and discretization methods.

Items	Discretization Methods
Pressure–Velocity Coupling	SIMPLE
Gradient	Least Squares Cell Based
Pressure	PRESTO!
Volume Fraction	Geo-Reconstruction
Momentum	First Order Upwind
Energy	First Order Upwind

**Table 4:** Comparison of simulation and experimental results for capillary tube inlet mass flow and external wall surface temperature of an ADN-based thruster.

	Temperature of Measurement Point (K)	Inlet Mass Flow (g/s)
Simulation	420.1	0.0300
Experiment	398.7	0.0308

**Table 5:** Comparison of simulation and experimental results for capillary tube inlet mass flow and external wall surface temperature of an ADN-based thruster.

	Heat Reflux Temperature (K)	Inlet Pressure (MPa)	Outlet Pressure (MPa)	Gravitational Acceleration (m/s^2^)
Case 1	400	1.5	1.0	0
Case 2	500	1.5	1.0	0
Case 3	600	1.5	1.0	0
Case 4	700	1.5	1.0	0
Case 5	800	1.5	1.0	0

## Data Availability

Not applicable.
